# Supplementary material for: Understanding Antimicrobial Resistance from the Perspective of Public Policy: A Multinational Knowledge, Attitude, and Perception Survey to Determine Global Awareness
Source: Antibiotics (Basel). 2021 Dec 4;10(12):1486. doi: 10.3390/antibiotics10121486 (PMC8698787; doi:10.3390/antibiotics10121486)
Supplement: Supplementary file 1 [file antibiotics-10-01486-s001.zip › Supplementary file 1.pdf]

Supplementary file 1: English version of the survey

Q1: How would you best describe your current position?

a. Executive role / decision making
b. Policy advisor
c. Other, namely: ……………….

Q2: Which level of governance are you currently involved in?

a. National / Cabinet
b. Provincial / District / Autonomous region
c. Regional / State
d. Municipal / Township
e. Non-government

Q3: Please specify (name of province/name of municipality/name of ministry):

…………………………………………………………..

Q4: How long have you been working at the current job?

a. < 1 year
b. 1 to 3 years
c. 3 to 5 years
d. 5 to 10 years
e. > 10 years

Q5: In which country are you currently working?

…………………………………………………………..

Q6:What is your nationality?

…………………………………………………………..

Q7: What is your highest level of education?

a. No formal education
b. High school
c. Vocational training
d. Bachelor degree
e. Master degree
f. Doctorate / PhD
g. Other, please specify: ……………………………………………

Q8: What best describes your field of expertise (education and/or occupation)?

a. Health-care (medicine, veterinary, nursing)
b. Life sciences (biology, chemistry, biomedical science)

c. Social sciences (economics, psychology, sociology, politics)
d. Humanities (law, arts, history, language, philosophy)
e. Information and technology (computer science, data science, engineering)
f. Business and management
g. Other, specify: ………………………………………………………………..

Q9: Please specify your age:
…………………………………………………………..

Q10: Please specify your gender:
a. Male
b. Female
c. Other / Prefer not to answer

Q11. Where do you reside?
a. Urban environment
b. Rural environment
c. Other,specify:

Q12. What is your political view (instead of affiliation)?
a. Left-Wing
b. Middle-left
c. Centre
d. Middle-right
e. Right-Wing
f. Prefer not to answer

Q12: Are you a member of a political party?
a. Yes
b. No

Q13: Which political party are you affiliated with?
a. Not applicable/I am not member of a party
b. Prefer not to answer
c. Yes. I am affiliated with a political party, namely:

Q14: Please select what best describes your opinion on the following issues. Please leave any of the subquestions open if you prefer not to answer the question, or don't know the answer.

	Strongly disagree	Disagree	Neutral	Agree	Strongly agree

1, In 30 years, antibiotic resistance will be responsible for more deaths than cancer					
2, Antibiotics can be used to treat viral infections					
3, Emerging antibiotic-resistant bacteria present in other countries or other continents will never become a problem in my country					
4, Unnecessary and inappropriate use of antibiotics in animal husbandry can negatively affect human health					
5, Antibiotic-resistant bacteria and antibiotic residues can spread via the environment and contribute to the antibiotic resistance issue					
6, It is easy to discover and produce new antibiotics					
7, Standard hygiene is essential to tackle antibiotic resistance					

Q15: Please select what best describes your opinion on the following issues. Please leave any of the subquestions open if you prefer not to answer the question, or don't know the answer.

	Strongly disagree	Disagree	Neutral	Agree	Strongly agree
1, I always finish my antibiotic treatment					
2, I take antibiotics quite often (i.e., more than once in 3 years)					
3, I am familiar with antibiotic resistance and aware of the associated problems for public health					
4, I do not believe that antibiotic resistance can become a health emergency issue					
5, Hospitals, veterinary clinics, and pharmaceutical industries are the actors responsible for antibiotic resistance and should solve the problem on their own					
6, The current coronavirus outbreak has increased my awareness of public health and the role of government in outbreak prevention and preparedness					

Q15: Please select what best describes your opinion on the following issues. Please leave any of the subquestions open if you prefer not to answer the question, or don't know the answer.

	Strongly disagree	Disagree	Neutral	Agree	Strongly agree
1, Interventions tackling antibiotic resistance have been implemented in the last 3 years					
2, A national antibiotic resistance action plan has been implemented in my country					
3, There are interventions in place in my country that target to reduce antibiotic resistance in both human and animals					
4, Antibiotic resistance needs governmental funding and effort in order to minimize the long-term effects					
5, Plans to tackle antibiotic resistance are mostly focussing on human health (i.e., human consumption of antibiotics) and are not including the livestock contribution					
6, Each medical facility has established antibiotic stewardship programs					
7, A surveillance program for antibiotic resistance bacteria has been established					
8, Tackling antibiotic resistance at the level of wastewater treatment is an upcoming idea in my country					
9, Antibiotic resistance is becoming a more popular topic in policies and regulations in my country					
10, Hospitals in my region are already taking measures to tackle AMR (have a dedicated budget for this)					
11, Hospitals have the initiative and show willingness to take action, but they need public money that is not available (yet)					
12, The funding resources to tackle antibiotic resistance have been increased in recent years and are prospected to increase more in the future					
13, One health approach should be used in designing antibiotic resistance surveillance and interventions					

Q18: What are the major achievements (in your area of governance) regarding the control of AMR, and what is the future plan to improve the current AMR policy?
………………………………………………………………..

Q19: What are the major challenges (in your area of governance) regarding antibiotic resistance?
………………………………………………………………..

Q20: Could you mention any specific project, initiative and/or measure to tackle AMR that has been already conducted/ implemented (in your area of governance)?
………………………………………………………………..

Q21: Please describe the financial resources / funding for these projects/initiatives (please indicate whether these projects are funded by (private, semi-private or public money) and from where (national, international, regional).
………………………………………………………………..
