# Supplementary material for: Understanding Antimicrobial Resistance from the Perspective of Public Policy: A Multinational Knowledge, Attitude, and Perception Survey to Determine Global Awareness
Source: Antibiotics (Basel). 2021 Dec 4;10(12):1486. doi: 10.3390/antibiotics10121486 (PMC8698787; doi:10.3390/antibiotics10121486)
Supplement: Supplementary file 1 [file antibiotics-10-01486-s001.zip › Supplementary file 2.pdf]

Supplementary file 2: Overview of invites sent per country and per governance level

Country	Number of invites sent
**Australia**	
National government	144
Regional government (Queensland)	117
**Belgium**	
National government	144
**Canada**	
National government	91
**Curaçao**	
National government	21
**India**	
Stakeholders (AMR insight)	47
**Israel**	
National government	120
**Mexico**	
Stakeholders (AMR insight)	2
**Morocco**	
National government	25
**Myanmar**	
Various (government and non-government) [A]	50
**The Netherlands**	
National government	225
Provincial government	570
Regional government (water boards) [A]	21
Regional government (municipalities) [A]	352
**Nigeria**	
National government	92
Stakeholders (AMR insight)	23
**Spain**	
Various (government and non-government) [A]	30
**Singapore**	

National government	272
**Surinam**	
National government	51

[A] Emails were sent to general email addresses or contact persons that were particularly requested to distribute the survey within their organisation and/or network. The total number of emails does therefore not represent the total number of individuals that were reached out to.
