# Supplementary material for: Understanding Antimicrobial Resistance from the Perspective of Public Policy: A Multinational Knowledge, Attitude, and Perception Survey to Determine Global Awareness
Source: Antibiotics (Basel). 2021 Dec 4;10(12):1486. doi: 10.3390/antibiotics10121486 (PMC8698787; doi:10.3390/antibiotics10121486)
Supplement: Supplementary file 1 [file antibiotics-10-01486-s001.zip › Supplementary file 4.pdf]

Variable	N	Good score [A] N (%)	OR 95% CI	Sig	aOR 95% CI	Sig	Fair score [A] N (%)	OR 95% CI	Sig	aOR 95% CI	Sig
	351	156 (41.3%)	-	-			74.1% (260)	-	-		
**Gender** [B]											
Female	182	44.0% (88)	ref		ref		72.5% (132)	ref		ref	
Male	163	44.2% (72)	1.01 [0.66 - 1.54]	0,968	1.06 [0.66 - 1.69]	0.805	69.9% (114)	0.88 [0.55 - 1.41]	0.596	0.96 [0.57 - 1.64]	0.892
**Age group** [B]											
< 40	94	41.5% (39)	ref		ref		71.3% (67)	ref		ref	
40 - 60	179	49.2% (88)	1.36 [0.82 - 2.26]	0,228	2.12 [1.11 - 4.04]	**0,023**	77.1% (138)	1.36 [0.77 - 2.39]	0.292	1.76 [0.85 - 3.64]	0.125
> 60	78	37.2% (29)	0.83 [0.45 - 1.55]	0,565	2.03 [0.91 - 4.52]	0,080	60.3% (47)	0.61 [0.32 - 1.15]	0.129	1.68 [0.70 - 4.01]	0.243
**Country class** [B]											
HIC	281	42.4% (119)	ref		ref		70.5% (198)	ref		ref	
LMIC	70	52.9% (37)	1.53 [0.90 - 2.58]	0,115	1.97 [0.84 - 4.62]	0,117	77.1% (54)	1.41 [0.77 - 2.61]	0.268	1.47 [0.52 - 4.10]	0.467
**Nationality** [C]											
The Netherlands	171	43.3% (73)	ref		ref		64.9% (111)	ref		ref	
Spain	97	39.2% (38)	0.84 [0.51 - 1.40]	0,513	0.44 [0.23 - 0.86]	**0,017**	78.4% (76)	1.96 [1.10 - 3.48]	**0.023**	1.07 [0.51 - 2.25]	0.856
Myanmar	34	26.5% (9)	0.47 [0.21 - 1.07]	0,073	0.65 [0.22 - 1.92]	0,432	64.7% (22)	0.99 [0.46 - 2.14]	0.982	1.39 [0.42 - 4.64]	0.593
**Duration** [B]											
< 3 years	133	45.1% (60)	ref				71.4% (95)	ref		ref	
3 - 10 years	129	44.2% (57)	0.96 [0.59 - 1.57]	0,880	0.86 [0.50 - 1.48]	0,593	72.1% (93)	1.03 [0.60 - 1.77]	0.905	0.90 [0.48 - 1.67]	0.728

> 10 years	89	43.8% (39)	0.95 [0.44 - 1.63]	0,849	0.66 [0.35 - 1.21]	0,178	71.9% (64)	1.02 [0.56 - 1.86]	0.938	0.65 [0.32 - 1.33]	0.239
**Education** [B]											
Master / PhD	157	54.8% (86)	ref		ref		79.6% (125)	ref		ref	
Bachelor	143	41.3% (59)	0.58 [0.37 - 0.92]	**0,020**	0.61 [0.37 - 0.99]	0,045	75.5% (108)	0.79 [0.46 - 1.36]	**0.396**	0.86 [0.48 - 1.53]	0.597
Lower levels	49	22.5% (11)	0.24 [0.11 - 0.50]	**<0.001**	0.25 [0.11 - 0.57]	**<0,001**	38.8% (19)	0.16 [0.08 - 0.32]	**<0.001**	0.16 [0.07 - 0.37]	**<0.001**
**Expertise** [B]											
Scientific	163	55.2% (90)	ref		ref		83.4% (136)	ref		ref	
Other	188	35.1% (66)	0.44 [0.29 - 0.67]	**<0.001**	0.49 [0.31 - 0.79]	**0.004**	61.7% (116)	0.32 {0.19 - 0.53]	**<0.001**	0.34 [0.19 - 0.56]	**<0.001**
**Living condition** [B]											
(Sub)urban	220	48.2% (106)	ref		ref		75.9% (167)	ref		ref	
Rural	130	37.7% (49)	0.65 [0.42 - 1.01]	0,057	0.79 [0.47 - 1.31]	0,357	64.6% (84)	0.58 [0.36 - 0.93]	**0,024**	0.73 [0.41 - 1.30]	0.287
**Occupation** [B]											
Government	303	37.3% (113)	ref				71.6% (217)	ref		ref	
Non-government	45	44.4% (20)	1.02 [0.54 - 1.92]	0,945	0.56 [0.23 - 1.34]	0.190	71.1% (32)	0.98 [0.49 - 1.95]	0.944	0.52 [0.18 - 1.46]	0.213
**Detailed occup.** [D]											
Municipal/ regional	183	38.3% (70)	ref				66.1% (121)	ref		ref	
Province	64	50.0% (32)	1.61 [0.91 - 2.86]	0,102	1.27 [0.69 - 2.36]	0.441	76.6% (49)	1.67 [0.87 - 3.22]	0,123	1.28 [0.62 - 2.68]	0.504
National	25	56.0% (14)	2.05 [0.88 - 4.78]	0,095	1.50 [0.57 - 3.95]	0,409	80.0% (20)	2.05 [0.73 - 5.72]	0,171	1.28 [0.40 - 4.12]	0.680
Non government	79	50.6% (40)	1.66 [0.97 - 2.82]	0,063	1.10 [0.51 - 2.39]	0,804	78.5% (62)	1.87 [1.01 - 3.47]	**0,047**	1.27 [0.47 - 3.44]	0.644

[A] Missing and unknown was not shown in the table, and total count does therefore not always equal 351.

[B] Multivariate analysis based on gender, age group, time at current role (duration), country class (HIC or LMIC), living condition, education, field of expertise and occupation (government or non-government)
[C] Similar as for B, excluding country class (HIC or LMIC)
[D] Similar as for B, excluding occupation (government or non-government)
