# Supplementary material for: Understanding Antimicrobial Resistance from the Perspective of Public Policy: A Multinational Knowledge, Attitude, and Perception Survey to Determine Global Awareness
Source: Antibiotics (Basel). 2021 Dec 4;10(12):1486. doi: 10.3390/antibiotics10121486 (PMC8698787; doi:10.3390/antibiotics10121486)
Supplement: Supplementary file 1 [file antibiotics-10-01486-s001.zip › Supplementary file 5.pdf]

Variable	N	Good AP [A] N (%)	OR 95% CI	Sig	aOR 95% CI	Sig	Fair AP [A] N (%)	OR 95% CI	Sig	aOR 95% CI	Sig
	351	41.6% (146)	-	-			83.8% (294)	-	-		
**Gender** [B]											
Female	182	62.1% (113)	ref		ref		81.3% (148)	ref		ref	
Male	163	55.2% (90)	0.75 [0.49 - 1.16]	0.196	0.76 [0.47 - 1.21]	0.246	85.9% (140)	1.40 [0.78 - 2.49]	0.255	1.57 [0.80 - 3.09]	0.194
**Age group** [B]											
< 40	94	45.7% (43)	ref		ref		71.3% (67)	ref		ref	
40 - 60	179	64.8% (116)	2.18 [1.31 - 3.63]	**0.003**	1.48 [0.78 - 2.78]	0.227	90.5% (162)	3.84 [1.96 - 7.51]	**<0.001**	2.38 [0.96 - 5.90]	0.062
> 60	78	59.0% (46)	1.70 [0.93 - 3.13]	0.085	1.85 [0.84 - 4.11]	0.129	83.3% (65)	2.01 [0.96 - 4.24]	0.065	2.46 [0.78 - 7.76]	0.125
**Country class** [B]											
HIC	281	63.0% (177)	ref		ref		88.3% (248)	ref		ref	
LMIC	70	40.0% (28)	0.39 [0.23 - 0.67]	**<0.001**	0.33 [0.14 - 0.75]	**0.009**	65.7% (46)	0.26 [0.14 - 0.47]	**<0.001**	0.19 [0.06 - 0.60]	**0.005**
**Nationality** [C]											
The Netherlands	171	56.7% (97)	ref		ref		85.4% (146)	ref		ref	
Spain	97	72.2% (70)	1.98 [1.16 - 3.38]	**0.013**	1.77 [0.90 - 3.45]	0.100	92.8% (90)	2.20 [0.91 - 5.30]	0.078	1.87 [0.63 - 5.58]	0.259
Myanmar	34	14.7% (5)	0.13 [0.05 - 0.36]	**<0.001**	0.15 [0.05 - 0.52]	**0.003**	38.2% (13)	0.11 [0.05 - 0.24]	**<0.001**	0.15 [0.04 - 0.57]	**0.005**
**Duration** [B]											
< 3 years	133	60.2% (80)	ref		ref		82.7% (110)	ref		ref	
3 - 10 years	129	55.8% (72)	0.84 [0.51 - 1.37]	0.477	1.06 [0.61 - 1.84]	0.832	83.7% (108)	1.08 [0.56 - 2.06]	0.826	1.82 [0.81 - 4.09]	0.148

> 10 years	89	59.6% (53)	0.98 [0.56 - 1.69]	0.929	0.71 [0.38 - 1.33]	0.288	85.4% (76)	1.22 [0.58 - 2.56]	0.595	0.89 [0.36 - 2.19]	0.800
**Education** [B]											
Master / PhD	157	61.2% (96)	ref		ref		88.5% (139)	ref		ref	
Bachelor	143	59.4% (85)	0.93 [0.32 - 1.16]	0.763	0.85 [0.51 - 1.43]	0.547	82.5% (118)	0.61 [0.32 - 1.18]	0.140	0.45 [0.21 - 0.97]	**0.043**
Lower levels	49	49.0% (24)	0.61 [0.32 - 1.16]	0.133	0.47 [0.22 - 0.99]	**0.048**	75.5% (37)	0.40 [0.18 - 0.90]	**0.027**	0.18 [0.06 - 0.53]	**0.002**
**Expertise** [B]											
Scientific	163	67.5% (110)	ref		ref		91.4% (149)	ref		ref	
Other	188	50.5% (95)	0.49 [0.32 - 0.76]	**0.001**	0.37 [0.23 - 0.62]	**<0.001**	77.1% (145)	0.32 [0.17 - 0.60]	**<0.001**	0.23 [0.10 - 0.50]	**<0.001**
**Living condition** [B]											
(Sub)urban	220	59.1% (130)	ref		ref		82.7% (182)	ref		ref	
Rural	130	57.7% (75)	0.94 [0.61 - 1.47]	0.798	0.72 [0.43 - 1.21]	0.211	85.4% (111)	1.22 [0.67 - 2.22]	0.516	0.74 [0.33 - 1.63]	0.451
**Occupation** [B]											
Government	303	61.4% (186)	ref		ref		86.8% (263)	ref		ref	
Non-government	45	18.0% (40)	0.42 [0.22 - 0.80]	**0.008**	0.73 [0.30 - 1.75]	0.483	62.2% (28)	0.25 [0.13 - 0.50]	**<0.001**	0.43 [0.14 - 1.27]	0.127
**Detailed occup.** [D]											
Municipal / regional	183	61.8% (113)	ref		ref		88.5% (162)	ref		ref	
Province	64	60.9% (39)	0.97 [0.54 - 1.73]	0.909	0.79 [0.43 - 1.48]	0.453	89.1% (57)	1.06 [0.43 - 2.61]	0.907	0.54 [0.19 - 1.50]	0.237
National	25	60.0% (15)	0.93 [0.40 - 2.18]	0.866	1.21 [0.44 - 3.30]	0.715	88.0% (22)	0.95 [0.26 - 3.45]	0.939	0.70 [0.15 - 3.22]	0.644
Non government	79	48.1% (38)	0.57 [0.34 - 0.98]	**0.041**	0.88 [0.38 - 2.03]	0.765	67.1% (53)	0.26 [0.14 - 0.51]	**<0.001**	0.18 [0.05 - 0.61]	**0.006**

[A] Missing and unknown was not shown in the table, and total count does therefore not always equal 351.

[B] Multivariate analysis based on gender, age group, time at current role (duration), country class (HIC or LMIC), living condition, education, field of expertise and occupation (government or non-government)

[C] Only participants from the Netherlands, Spain and Myanmar were included. Multivariate analysis similar as for B, excluding country class (HIC or LMIC)

[b] Similar as for B, excluding occupation (government or non-government)
