# Supplementary material for: Understanding Antimicrobial Resistance from the Perspective of Public Policy: A Multinational Knowledge, Attitude, and Perception Survey to Determine Global Awareness
Source: Antibiotics (Basel). 2021 Dec 4;10(12):1486. doi: 10.3390/antibiotics10121486 (PMC8698787; doi:10.3390/antibiotics10121486)
Supplement: Supplementary file 1 [file antibiotics-10-01486-s001.zip › Supplementary file 6.pdf]

Supplementary file 3: Univariate (odds ratio, OR) and multivariate analysis (adjust odds ratio, aOR) of political KAP

Variable	N	Good score [A] N (%)	OR 95% CI	Sig	aOR 95% CI	Sig	Fair score [A] N (%)	OR 95% CI	Sig	aOR 95% CI	Sig
	351	4.6% (16)	-	-			19.7% (69)	-	-		
**Gender** [B]											
Female	182	2.8% (5)	ref		ref		17.0% (31)	ref		ref	
Male	163	6.8% (11)	2.56 [0.87 - 7.54]	0.088	2.32 [0.73 - 7.41]	0.156	21.5% (35)	1.33 [0.78 - 2.28]	0.296	1.36 [0.77 - 2.41]	0.293
**Age group** [B]											
< 40	94	5.3% (5)	ref		ref		24.5% (23)	ref		ref	
40 - 60	179	2.2% (7)	0.41 [0.11 - 1.55]	0.188	0.42 [0.08 - 2.13]	0.298	16.8% (30)	0.62 [0.34 - 1.15]	0.128	0.61 [0.29 - 1.30]	0.201
> 60	78	9.0% (4)	1.75 [0.53 - 5.75]	0.354	1.69 [0.30 - 9.69]	0.555	20.5% (16)	0.80 [0.39 - 1.64]	0.538	0.82 [0.33 - 2.08]	0.682
**Country class** [B]											
HIC	281	4.3% (12)	ref		ref		18.9% (53)	ref		ref	
LMIC	70	5.7% (4)	1.36 [0.42 - 4.35]	0.606	3.55 [0.57 - 22.29]	0.176	22.9% (16)	1.27 [0.68 - 2.40]	0.452	1.38 [0.53 - 3.59]	0.515
**Nationality** [C]											
The Netherlands	171	6.4% (11)	ref		ref		22.2% (38)	ref		ref	
Spain	97	1.0% (1)	0.15 [0.02 - 1.19]	0.073	0.18 [0.02 - 1.80]	0.145	13.4% (13)	0.54 [0.27 - 1.08]	0.080	0.47 [0.20 - 1.10]	0.083
Myanmar	34	2.9% (1)	0.44 [0.06 - 3.53]	0.440	1.40 [0.10 - 19.86]	0.805	20.6% (7)	0.91 [0.37 - 2.25]	0.834	1.25 [0.36 - 4.29]	0.722
**Duration** [B]											

< 3 years	133	6.8% (9)	ref		ref		20.3% (27)	ref		ref	
3 - 10 years	129	1.6% (2)	0.22 [0.05 - 1.02]	0.054	0.20 [0.04 - 1.05]	0.058	19.4% (25)	0.94 [0.51 - 1.73]	0.852	0.94 [0.48 - 1.81]	0.844
> 10 years	89	5.6% (5)	0.82 [0.27 - 2.53]	0.730	0.86 [0.24 - 3.07]	0.817	19.1% (17)	0.93 [0.47 - 1.82]	0.826	1.04 [0.49 - 2.20]	0.913
**Education** B											
Master / PhD	157	5.7% (9)	ref		ref		23.6% (37)	ref		ref	
Bachelor	143	2.1% (3)	0.35 [0.09 - 1.33]	0.123	0.48 [0.12 - 1.96]	0.304	16.8% (24)	0.65 [0.37 - 1.16]	0.146	0.64 [0.35 - 1.18]	0.153
Lower levels	49	8.2% (4)	1.46 [0.43 - 4.97]	0.543	1.00 [0.23 - 4.37]	0.996	16.3% (8)	0.63 [0.27 - 1.47]	0.287	0.58 [0.23 - 1.49]	0.261
**Expertise** B											
Scientific	163	6.1% (10)	ref		ref		23.3% (38)	ref		ref	
Other	188	3.2% (6)	0.50 [0.18 - 1.42]	0.195	0.39 [0.12 - 1.24]	0.110	16.5% (31)	0.65 [0.38 - 1.10]	0.110	0.67 [0.38 - 1.19]	0.170
**Living condition** B											
(Sub)urban	220	3.6% (8)	ref		ref		17.7% (39)	ref		ref	
Rural	130	6.2% (8)	1.74 [0.64 - 4.75]	0.281	1.94 [0.54 - 6.92]	0.309	22.3% (29)	1.33 [0.78 - 2.28]	0.296	1.68 [0.90 - 3.13]	0.103
**Occupation** B											
Government	303	5.0% (15)	ref		ref		19.5% (59)	ref		ref	
Non-government	45	2.2% (1)	0.44 [0.06 - 3.39]	0.428	0.19 [0.02 - 2.05]	0.172	17.8% (8)	0.89 [0.40 - 2.02]	0.788	0.63 [0.22 - 1.78]	0.381
**Detailed occup.** D											
Municipal/ regional	183	4.9% (9)	ref		ref		22.4% (41)	ref		ref	
Province	64	4.7% (3)	0.95 [0.25 - 3.63]	0.941	0.89 [0.21 - 3.84]	0.879	12.5% (8)	0.49 [0.22 - 1.12]	0.092	0.43 [0.18 - 1.02]	0.055
National	25	4.0% (1)	0.81 [0.10 - 6.64]	0.841	0.30 [0.02 - 5.16]	0.404	16.0% (4)	0.66 [0.21 - 2.03]	0.458	0.45 [0.12 - 1.67]	0.232
Non government	79	3.8% (3)	0.76 [0.20 - 2.90]	0.691	0.28 [0.03 - 3.18]	0.307	20.3% (16)	0.88 [0.46 - 1.68]	0.699	0.62 [0.23 - 1.71]	0.360

[A] Missing and unknown was not shown in the table, and total count does therefore not always equal 351.

[B] Multivariate analysis based on gender, age group, time at current role (duration), country class (HIC or LMIC), living condition, education, field of expertise and occupation (government or non-government)

[C] Similar as for B, excluding country class (HIC or LMIC)

[D] Similar as for B, excluding occupation (government or non-government)
